# Impact of long-term care facilities’ size on adherence to COVID-19’ infection prevention guidance

**DOI:** 10.1590/1518-8345.5581.3516

**Published:** 2022-04-29

**Authors:** Patrick Alexander Wachholz, Ruth Caldeira de Melo, Alessandro Ferrari Jacinto, Paulo José Fortes Villas Boas

**Affiliations:** 1 Universidade Estadual Paulista, Faculdade de Medicina de Botucatu, Botucatu, SP, Brasil.; 2 Universidade de São Paulo, Escola de Artes, Ciências e Humanidades, São Paulo, SP, Brasil.; 3 Universidade Federal de São Paulo, Escola Paulista de Medicina, São Paulo, SP, Brasil.

**Keywords:** Aged, COVID-19, Guideline Adherence, Long-Term Care, Coronavirus, Homes for the Aged, Idoso, COVID-19, Fidelidade a Diretrizes, Assistência de Longa Duração, Coronavirus, Instituição de Longa Permanência para Idosos, Anciano, COVID-19, Adhesión a Directriz, Cuidados a Largo Plazo, Coronavírus, Hogares para Ancianos

## Abstract

**Objective:**

to evaluate the adherence of Brazilian long-term care facilities to the World Health Organization Infection Prevention and Control guidance, and assess the association of their size with the adherence to these recommendations.

**Method:**

cross-sectional study conducted with facilities’ managers. Authors developed a 20-item questionnaire based on this guidance, and a global score of adherence, based on the adoption of these recommendations. Adherence was classified as (1) excellent for those who attended ≥14 out of 20 recommendations; (2) good for 10 to 13 items; and (3) low for those with less than ten items. Facilities’ sizes were established as small, intermediate, and large according to a two-step cluster analysis. Descriptive statistics and chi-square tests were used at a 5% significance level.

**Results:**

among 362 included facilities, 308 (85.1%) adhered to 14 or more recommendations. Regarding its size, adherence to screening COVID-19 symptoms of visitors (p=0.037) and isolating patients until they have had two negative laboratory tests (p=0.032) were lower on larger ones compared to medium and small facilities.

**Conclusion:**

adherence to COVID-19 mitigation measures in Brazilian facilities was considered excellent for most of the recommendations, regardless of the size of the units.

## Resumo

**Objetivo:**

avaliar a adesão das instituições de longa permanência brasileiras às orientações de Prevenção e Controle de Infecções da Organização Mundial da Saúde, observando a associação entre seu porte e a adesão a essas recomendações.

**Método:**

estudo transversal realizado com gestores de estabelecimentos. Os autores desenvolveram um questionário de 20 itens, com base nessas orientações, e um escore global de adesão, com base na adesão a essas recomendações. A adesão foi classificada como (1) excelente para aquelas que atenderam ≥14 de 20 recomendações, (2) bom para 10 a 13 itens e (3) baixo para aquelas com menos de dez itens. O tamanho das instalações foi classificado como pequeno, médio e grande, de acordo com uma análise de cluster em duas etapas. Estatística descritiva e teste de qui-quadrado foram utilizados com nível de significância de 5%.

**Resultados:**

das 362 instituições incluídas, 308 (85,1%) aderiram a 14 ou mais recomendações. Em relação ao seu tamanho, a adesão à triagem de sintomas de COVID-19 dos visitantes (p=0,037) e ao isolamento de pacientes até que tenham dois exames laboratoriais negativos (p=0,032) foi menor em estabelecimentos maiores, em comparação com estabelecimentos de médio e pequeno porte.

**Conclusão:**

a adesão às medidas de mitigação da COVID-19 nas unidades brasileiras foi considerada excelente para a maioria das recomendações, independentemente do porte das unidades.

## Resumen

**Objetivo:**

evaluar la adhesión de instituciones brasileñas de larga estancia a las orientaciones de Prevención y Control de Infecciones de la Organización Mundial de la Salud y evaluar la asociación entre su tamaño y la adhesión a esas recomendaciones.

**Método:**

estudio transversal realizado con gerentes de establecimientos. Los autores desarrollaron un cuestionario de 20 ítems basado en estas directrices y un puntaje general de cumplimiento acorde a la observancia de estas recomendaciones. La adhesión se calificó (1) excelente para aquellas que cumplieron con ≥14 de 20 recomendaciones; (2) buena para 10 a 13 artículos; y (3) baja para aquellas con menos de diez elementos. El tamaño de las instalaciones se clasificó como pequeños, medianos y grandes de acuerdo con un análisis de clúster de dos pasos. Se utilizó estadística descriptiva y la prueba de chi-cuadrado con un nivel de significancia del 5%.

**Resultados:**

de las 362 instituciones incluidas, 308 (85,1%) se adhirieron a 14 o más recomendaciones. En cuanto a su tamaño, la adhesión al cribado de síntomas de COVID-19 de los visitantes (p=0,037) y al aislamiento de los pacientes hasta que tengan dos pruebas de laboratorio negativas (p=0,032) fue menor en los establecimientos más grandes en comparación con los establecimientos medianos y pequeños.

**Conclusión:**

la adhesión a las medidas de mitigación de la COVID-19 en las unidades brasileñas fue considerada excelente para la mayoría de las recomendaciones, independientemente del tamaño de las unidades.

**Highlights:**

(1) Prevention is a critical component of infection control during emerging infections.

(2) COVID-19 disproportionately affected the long-term care facilities (LTCF).

(3) The preparedness for mitigating COVID-19 in Brazilian LTCF was considered excellent.

(4) Adherence to COVID-19 infection prevention and control (IPC) measures was not influenced by the size of the facilities.

(5) Difficulties were mostly related to financial distress and managing the workforce

## Introduction

Older people living in long-term care facilities (LTCF) have been disproportionately affected by the COVID-19 pandemic^1^ ^-^ ^6^. The pandemic overwhelmed health systems worldwide and placed a spotlight on the weaknesses (or absence) of national datasets and of infection prevention and control (IPC) practices for LTCF^7^. 

The prevalence of COVID-19 in the community is a strong predictor of the number of cases and deaths in LTCF^4^ ^,^ ^8^. Mortality rates were markedly increased among the most frail residents, just like for those living in more crowded facilities^9^. Moreover, the probability for COVID-19 cases seems to be higher among large, non-for-profit, and metropolitan county facilities^4^. Corroborating these findings, the characteristics of North-American LTCFs were examined with documented COVID-19 cases and it was found that infections were related to facility location (i.e, urban) and size (i.e, with 50 beds or more)^1^.

On the other hand, other authors showed that high nurse aides and total nursing hours contribute to mitigating the COVID-19 infection in LTCF^4^. High staffing ratios^9^ and early and robust IPC practices^8^ seem to be associated with low COVID-19 cases and death rates among older adults living in these settings.

Early on, the World Health Organization (WHO), the Centers for Disease Control (CDC), and regional organizations had published recommendations and policy actions to mitigate the impact of COVID-19 on people who rely on the long-term care sector^10^ ^-^ ^13^. To the best of our knowledge, however, the COVID-19 preparedness in LTCF and the adherence to these recommendations have not been sufficiently investigated. 

A North-American small sampled study found that the CDC recommendations were most used (88%) IPC guidance, followed by state or local health departments (84%), and by the WHO guidance (48%)^14^. More than half of the managers (54%) had separate COVID-19 plans, almost all (96%) had policies for screening visitors, and most (68%) indicated they had a local referral hospital accepting their patients under investigation for COVID-19. Near to 83% expected significant staff shortages, while 66% reported access to COVID-19 testing^14^.

The implementation of evidence in clinical practices is neither easy nor fast. Barriers and facilitators need to be promptly and correctly addressed, particularly at the organizational level^15^ ^-^ ^17^. It is not clear if the sizes of LTCFs might be a barrier to implementing strategies to mitigate COVID-19 dissemination. For example, in larger LTCFs, the greater the number of residents, collaborators, and visitors, the greater the number of workers and training needs, the preparation of rotation schedules for abstaining due to potential leaves, and employment demands for turnover/retention, and the risk for a shortage of supplies^18^ ^-^ ^19^.

This study aimed to evaluate the adherence of Brazilian LTCF managers to the WHO IPC guidance and assess the association of their size with the adherence to the recommendations for COVID-19 mitigation.

## Method

### Ethical aspects

Ethical approval was provided by the institutional review board from the Botucatu Medical School, São Paulo State University - UNESP, for this cross-sectional study (CAAE 30577520.0.0000.0008, protocol No. 4.012.489), and all participants provided online informed consents. The datasets supporting the findings of this study are available^2^. For the purposes of this study, a LTCF provides long-term care and/or rehabilitative, restorative, and end-of-life care to residents in need of assistance with activities of daily living, including a variety of services (social, medical and personal care) to people who are unable to live independently. 

### Design and participants

This cross-sectional study was conducted exclusively through electronic platforms for twelve consecutive weeks from May 5^th^, 2020, using Google Forms. Managers from Brazilian LTCF for older people were the population of interest. We obtained their contacts through listings with domains available on the Internet, including active search with health secretariats, epidemiological surveillance, stakeholders, the Brazilian Unified Social Assistance System (SUAS), and LTCF support groups, including websites and social media groups. We did not apply restrictions regarding the size, location, or type of facility. 

### Data collection

The World Health Organization published the “Infection Prevention and Control Guidance for Long-Term Care Facilities in the Context of COVID-19” on March 21^st^, 2020, intending to guide IPC practices for this sector to prevent the virus from entering the facility and spreading within and outside the facility^10^. Initially prepared in English, the guidance was later translated into Portuguese by the Pan American Health Organization^10^. Unlike other assessment tools developed by WHO^20^ ^-^ ^21^, this interim guidance was thought to inform and support LTCF managers in implementing minimum requirements for IPC practices in the sector; it was not intended to be used as an audit tool but to help assess, plan, organize and implement IPC practices and activities in an early context of low availability of supporting evidence.

Based on the recommendations from the WHO guidance^10^ the authors developed a 20-item questionnaire, using multiple-choice and dichotomous questions. Since to date reliable national data on the sector is missing, twenty-six additional questions were included, aiming to provide information on LTCF characterization, and provision/availability of personal protective equipment, infrastructure for management of suspected/infected cases and deaths, and contingency plans for potential outbreaks. An open question about the most significant difficulties faced by the facility in tackling the pandemic was also included. The questionnaire was divided into nine sections: prevention, physical distance within the institution, rules for visitors, prospective surveillance for COVID-19 among residents, prospective surveillance among employees, source control, restrictions on movement and transportation, provision and availability of personal protective and cleaning equipment, technical support to face the pandemic.

The authors developed a global score of adherence to the 20 questions based on the original WHO guidance (Tables 2 and 3). For the purposes of this study, adherence was classified as (1) **excellent** for those LTCF that attended at least 14 items out of the 20 recommendations (i.e.,70%); (2) **good** for those LTCF that attended 10 to 13 items (i.e., 50 to 69%); and (3) **low** for those that attended less than ten items (i.e.,<49%). 

The estimated time to complete the questionnaire was 30 minutes. Respondents were free to participate more than once, but each LTCF was included only once in the study, having been chosen the most complete and recent answer for the analysis.

### Data treatment and analysis

Statistical analysis was performed using SPSS version 20. When respondents did not report data for one or more variables, we described them as missing, informing their proportional representation. As there is no standard definition for the LTCF size classification, for the purpose of this paper, a two-step cluster analysis was applied to the continuous variable “number of residents” using the automatic clustering algorithm in SPSS version 20. Three cluster groupings were established: small, intermediate and large facilities. Descriptive statistics and chi-square tests were used at a 5% significance level.

The open question was analyzed based on the thematic content analysis^22^, divided into three steps: pre-analysis, material exploration, and treatment of results. In the first step, a fluctuating reading was performed to identify the main difficulties faced by the LTCF. After that, the material was explored to establish central themes and related subcategories. The answers were codified, allowing the calculation of each category’s frequency and extraction of parts of the text related, creating a Venn diagram.

## Results

We received 374 contributions during the 12-week investigation period. Five managers answered the questionnaire twice; their oldest and incomplete responses were excluded, as well as one not-Brazilian LTCF answer. Six managers did not inform data on the number of residents, so their responses were not included. 

Altogether, 362 LTCF accommodated a population of 11 903 older adults. The Brazilian Southeast region concentrates the largest proportion of included facilities (53.8%), mostly for-profit (39.7%) and not-for-profit centers receiving government/non-government subsidies (37.5%). Table 1 presents an overview characterization of the sample, according to a three clustering LTCF size division.

**Table 1:** Characteristics of 362 Brazilian long-term care facilities for older people according to the facility’s size, whose managers answered an online questionnaire to identify their preparedness for the COVID-19 pandemic (n=362). Botucatu, SP, Brazil, 2020

Sample characteristics	Size of the facility		
	Small facilities (n= 172)	Intermediate facilities (n= 157)	Large facilities (n= 33)
**Number of Residents**			
Median (95%CI)	18 (16.8 - 18.5)	37 (37.5 - 40.3)	76 (74.6 - 92.2)
Mean (SD)	17.7 (5.7)	38.9 (8.8)	83.4 (24.8)
**Funding Source, n (%)**			
Public	10 (5.8)	6 (3.8)	2 (6.0)
Profit	98 (56.9)	40 (25.5)	4 (12.1)
Not-for-profit type 1^*^	43 (25.0)	76 (48.4)	18 (54.5)
Not-for-profit type 2^†^	21 (12.2)	35 (22.2)	9 (27.3)

^*^Long-term care facilities that receive government/non-government subsidies; ^†^Long-term care facilities that work on behalf of others than their founders or directors and can be reimbursed for their services (usually charity entities).

One hundred and sixty-six LTCF (45.1%), regardless of the type of funding, declared that they had not received subsidies or external financing to prepare and face the pandemic, including training, protective equipment purchase, and infrastructure adaptations for the respiratory isolation of suspected cases. 

Two hundred and thirty-five (64.9%; missing 74 answers) answered that their facilities already had the necessary infrastructure to accommodate COVID-19 suspected cases, including accommodation with individual bathrooms, with sufficient spaces to carry out preventive, hygiene, and protection measures for workers and residents. The availability of laboratory testing for influenza and coronavirus was low: 23.5% (n = 85) did not have access to both testing, and 17.4% (n=63) had access only to SARS-Cov-2 rapid test kits. 

Table 2 and 3 summarizes the adherence to the 20 items based on WHO IPC guidance in the sample. Three hundred and eight managers (85.1%) adhered to 14 or more IPC guidance recommendations; three were classified as low adherent, and 35 (9.7%) were missing cases. Regarding LTCF size, adherence to screening COVID-19 signs and symptoms of visitors (p=0.037) and isolating COVID-19 patients until they have two negative laboratory tests (p=0.032) were lower on larger ones compared to medium and small facilities. No significant differences were found for the other WHO’s guidance among the three sizes of LTCF studied.

**Table 2:** Adherence to the World Health Organization guidance for care homes in the context of COVID-19 in 362 Brazilian facilities (n=362). Botucatu, SP, Brazil, 2020

Recommendations	Adherence n (%)				
	Total(n=362)	Small (n=172)	Intermediate(n=157)	Large(n=33)	p-value
**Prevention**					
Provide COVID-19 IPC* training to all employees	288 (79.6)	132 (76.7)	128 (81.5)	28 (84.8)	0.611
Provide information sessions for residents	258 (71.3)	121 (70.3)	109 (69.4)	28 (84.8)	0.241
Regularly audit IPC* practices	320 (88.4)	146 (84.9)	145 (92.4)	29 (87.9)	0.108
According to local policies, provide annual vaccination	344 (95.0)	162 (93.6)	151 (96.2)	32 (97.0)	0.720
**Physical distance within the institution**					
Post reminders, posters, flyers around the facility	302 (83.4)	148 (86.0)	125 (79.6)	29 (87.9)	0.423
Physical distancing in the facility (for group activities)	305 (84.3)	146 (84.9)	132 (84.1)	27 (81.8)	0.894
Physical distancing in the facility (for meals)	212 (58.6)	103 (59.9)	92 (58.6)	17 (51.5)	0.772
Require residents and employees to avoid touching	344 (95.0)	163 (94.8)	148 (94.3)	33 (100)	0.539
**Rules for visitors**					
Screen for signs and symptoms or risk for COVID-19	337 (93.1)	161 (93.6)	148 (94.3)	28 (84.8)	0.037

*IPC = Infection prevention and control.

**Table 3:** Adherence to the World Health Organization guidance for care homes in the context of COVID-19 (n=362). Botucatu, SP, Brazil, 2020

Prospective surveillance among residents	Total (n=362)	Small (n=172)	Intermediate (n=157)	Large (n=33)	p-value
Assess the health status of any new residents at admission	344 (95.0)	164 (95.3)	148 (94.3)	32 (97.0)	0.700
Assess each resident twice daily for signs	336 (92.8)	160 (93.0)	146 (93.0)	30 (90.9)	0.206
Immediately report residents with fever or respiratory symptoms	358 (98.9)	168 (97.7)	157 (100)	33 (100)	0.346
**Prospective surveillance among employees**					
Prospective surveillance for employees (in case of symptoms)	355 (98.1)	168 (97.7)	156 (99.4)	31 (93.9)	0.223
Follow up on employees with unexplained absences	328 (90.6)	157 (91.3)	142 (90.4)	29 (87.9)	0.568
Undertake temperature checks for all employees at the facility entrance	275 (76.0)	128 (74.4)	122 (77.7)	25 (75.8)	0.777
**Availability of personal protective equipment**					
Employees should use contact and droplet precautions	327 (90.3)	155 (90.1)	143 (91.1)	29 (87.9)	0.792
PPE* usage following recommended procedures to avoid contamination	345 (95.3)	162 (94.2)	152 (96.8)	31 (93.9)	0.386
**Source control**					
Notify local authorities about any suspected case and isolate residents with the onset of respiratory symptoms	228 (63.0)	101 (59.7)	100 (43.9)	27 (81.8)	0.190
**Restrictions on movement and transportation**					
Confirmed patients should not leave their rooms while ill.	338 (93.4)	162 (94.2)	147 (93.6)	29 (87.9)	0.057
Isolate COVID-19 patients until they have two negative laboratory tests	329 (90.9)	158 (91.9)	142 (90.4)	29 (87.9)	0.032

*PPE = Personal protective equipment.

Table 4 describes the issues that could influence preparedness and adherence to additional technical support recommendations to face the pandemic of COVID-19 in LTCF regarding questions not included in the global adherence score. The most frequent issues identified were related to external support (74.3% did not receive support to plan and execute the training and contingency plans), followed by difficulties to acquire personal protective equipment for residents and employees (47.0%) and management of death cases (46.1%). A multidisciplinary planning committee, created specifically to deal with COVID-19 issues, was less frequent in small LTCF (49.4%) compared to intermediate (65.0) and large ones (75.8) (p=0.034).

**Table 4:** Preparedness and adherence to technical support recommendations to face the pandemic of COVID-19 in 362 Brazilian long-term care facilities (n=362). Botucatu, SP, Brazil, 2020

Recommendations	Adherence n (%)				
	Total (n=362)	Small (n=172)	Intermediate (n=157)	Large (n=33)	p-value
**Contingency plan**					
Managers discussed, analyzed, or considered creating a contingency plan to identify the minimum number of employees needed to operate safely.	276 (72.7)	128 (74.4)	124 (79.0)	24 (72.7)	0.838
Managers effectively implemented a contingency plan to identify the minimum number of employees needed to operate safely and how to hire or recruit them.	241 (66.6)	110 (64.0)	109 (69.4)	22 (66.7)	0.304
A multidisciplinary planning committee was created specifically to decide and discuss the planning of actions to prevent and combat COVID-19	212 (58.6)	85 (49.4)	102 (65.0)	25 (75.8)	0.034
**Personal protective equipment**					
Residents and employees have comfortable and sufficient access to PPE*	323 (89.2)	156 (90.7)	137 (87.3)	30 (90.9)	0.716
The facility is facing difficulties to buy or keep its cleaning supplies and PPE* for residents and employees.	192 (53.0)	89 (51.7)	89 (56.7)	14 (42.2)	0.499
**Management of suspected/infected cases and deaths**					
The facility has established care flows with local health authorities or referral units to transfer suspected cases.	228 (63.0)	101 (58.7)	100 (63.7)	27 (81.8)	0.199
Managers created a contingency plan to deal with death cases within the unit.	195 (53.9)	80 (46.5)	95 (60.5)	20 (60.6)	0.249

*PPE = Personal protective equipment

The analysis of the manager’s answers to the open question demonstrated that the majority (98%) of LTCF were having difficulties facing the COVID-19 pandemic. According to the managers, the most affected areas were related to maintaining sufficient materials (42%) (e.g., PPE, hygiene and clean, COVID-19 tests); financial distress (39%) (e.g., the extra cost with materials, the additional cost with staff, and fundraising issues); and manage the workforce (24%) (e.g., absence and replacement, awareness and protocol compliance, qualification and training, and emotional distress). Problems with infrastructure (i.e., adaptation for isolation suspected/confirmed case and enough room for maintenance of distancing procedures), and residents (e.g., awareness and protocol compliance, emotional distress, keep social distancing, management of dementia, social, and visits restriction and absence of family contact) were reported by 14% of the LTCF. 

**Figure 1:**
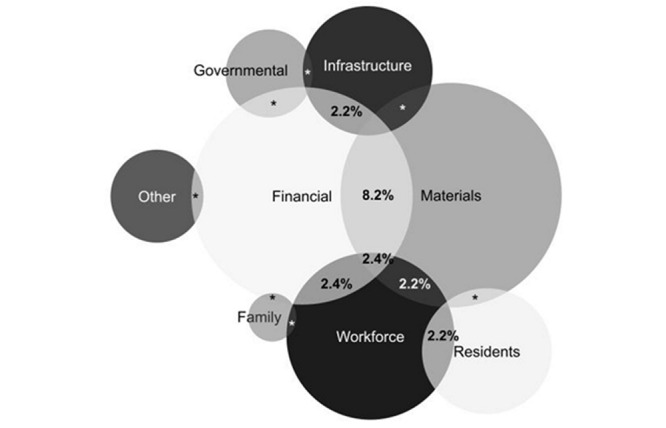
Venn diagram showing the overlap among the issues faced by the long-term care facilities. Botucatu, SP, Brazil, 2020

Figure 1 illustrates the overlap among the issues faced by the LTCF, according to their managers. The main overlapping detected was between financial and materials issues (8.2%), followed by financial vs. workforce (2.4%) and financial vs. materials vs. workforce (2.4%). A 2.2% of overlapping was also observed for financial vs. infrastructure, materials vs. workforce, and workforce vs. resident’s issues. The remaining combinations of issues faced by LTCF were present in lower than 2.0% of responses.

The symbol * indicates overlap of less than 2,0%

## Discussion

In this cross-sectional study, most Brazilian LTCFs (85.1%) reported an excellent adherence to the overall recommendations based on WHO IPC guidance to mitigate COVID-19, albeit 98% declared difficulties with a shortage of supplies, PPE and materials, financial problems, and managing the workforce. 

Although previous evidence clearly suggested that the size of LTCF was associated with an increased risk of outbreaks and deaths, and possibly with difficulties in adhering to IPC measures^1^ ^,^ ^4^, we found a high overall adherence rate regardless of their size. The global score was slightly smaller in larger LTCF for “screening external visitors”, and “isolating contaminated residents”. The adherence to the recommendation of establishing multidisciplinary committees to combat COVID-19 was lower as smaller were the LTCF.

Although the size of facilities has not been assessed in a recent systematic review^23^ on the epidemiology and clinical features of COVID-19 outbreaks in aged care facilities, larger facilities were previously found to be correlated with the spread of infection^23^ ^-^ ^24^. However, increasing testing capacity and updating surveillance protocols accordingly could facilitate earlier detection of emerging outbreaks, and help these units manage the supply of care workers and quality of nursing homes, especially their response to infectious diseases^24^ ^-^ ^25^.

Many LTCFs perceived a considerable workload during the COVID-19 pandemic. Tasks and activities that require direct involvement of the workforce, such as caring for infected residents (particularly those with functional impairment and cognitive decline) and screening external visitors, would be expected to have the most significant impact on facilities with the highest number of residents. This impact, however, was not sufficient to reduce the adherence to IPC recommendations significantly.

COVID-19 mortality was found to be higher in facilities with more significant crowding (9.7% vs. 4.5% in low crowding) in the U.S.A., regardless of the unit’s size^26^. In Canada, the size of LTCF was strongly associated with COVID-19 outbreaks (odds ratio *per* 20-bed increase 3·35, 95% CI 1·99-5·63)^27^. Meanwhile, in the U.K., the likelihood of spread was higher for larger LTCF (>20 beds) and when workers and facilities did not adhere to IPC measures to mitigate infection^28^. Preparedness and adherence to recommendations by LCTF, however, were poorly described in low- and middle-income countries (LMIC).

Prevention is a critical component of infection control, particularly during emerging infections. The long-term care sector in LMICs remains mostly underdeveloped, and specific strategies must be considered and suited to promote and successfully implement IPC protocols and guidelines. IPC implementation significantly differed among high-risk populations between higher - and lower -prevalence groups in the social distancing and PPE categories in a high-income country^26^.

Other factors may potentially influence the adherence to IPC, acting as facilitators or barriers to its implementation. The community prevalence of COVID-19, the availability of screening tests, and the rate of infection among workers (including staff turnover and retention) may influence the adherence to some of the IPC recommendations. The facility’s occupancy, inadequate staff IPC measures to minimize staff-to-staff transmission, delayed recognition of cases in residents because of a low index of suspicion, and residents at risk for severe morbidity and death sharing a location are also factors that may influence affect adherence^28^.

Data from previous works conducted with 23 896 Brazilian respondents (mean age: 47.4 years) found that participants showed a satisfactory level of adherence to national COVID-19 prevention guidelines. Younger people, males, persons living in a rural area/village or popular neighborhoods, students, and workers reported less preventive behavior^29^.

The results of this study have limitations inherent to the cross-sectional design adopted and to recall bias. Likewise, potential selection bias may have privileged LTCF with access to the internet and those with more complete teams, including workers dedicated to administrative activities. Although the questions were developed by the authors based on the WHO guidance, they were not intended to be an assessment or audit tool, and answers provided by LTCF managers may not reflect the real situation in the included facilities. Likewise, there are no cutoff points established to classify facilities by their size, which could influence the findings. Despite this, all the definitions adopted for the purpose of this study were defined *a priori*, and a large and nationally representative number of units were included.

COVID-19 has been a pandemic, or a syndemic^30^, of inequalities: countries with successful responses developed partnerships on multiple levels across government sectors, had timely triage and referral of suspected cases^31^. Part of the drama observed at the beginning of the pandemic when mortality by COVID-19 among residents was brutally high in some countries was partly due to the absence of official guides and regulations in natural disasters pandemics worldwide^32^. 

Few initiatives in Brazil have been dedicated to recommending best practices in COVID-19 mitigation in the LTC sector. Of the most representative ones, such as those led by the National Front for Strengthening the LTCF^33^ and the Brazilian Society of Geriatrics and Gerontology^34^, none so far has been investigated for its effectiveness. By identifying the adherence rate to IPC recommendations, LTCF managers and their workforce can potentially recognize the effectiveness of these actions, drawing and adapting better contingency plans that may allow the sector to be prepared for new emerging threats. Likewise, in practical terms, nursing staff from LTCF may find this script of questions useful when checking for best IPC practices in the sector.

## Conclusion

In conclusion, the adherence to the recommendations from WHO IPC guidance was considered excellent for most of the proposed items, regardless of the size of the units. Adherence to screening COVID-19 symptoms of visitors and isolating patients until they have two negative laboratory tests were the only aspects that were lower on larger facilities compared to medium and small ones. According to the managers, most of the ILPIs faced difficulties dealing with the COVID-19 pandemic, which are mainly related to financial difficulties, lack of personal protective equipment, and hygiene and cleaning 

## Footnotes

* Available from: https://doi.org/10.7910/DVN/LXBFQG
